# Termite Communities along A Disturbance Gradient in a West African Savanna

**DOI:** 10.3390/insects10010017

**Published:** 2019-01-08

**Authors:** Janine Schyra, Judith Korb

**Affiliations:** 1 Behavioral Biology, University of Osnabrueck, Barbarastr. 11, D-49076 Osnabrueck, Germany; judith.korb@biologie.uni-freiburg.de; 2 Evolution and Ecology, Albert-Ludwigs-University Freiburg, Hauptstr. 1, D-79104 Freiburg im Breisgau, Germany

**Keywords:** termite, community assembly, pest, disturbance, West Africa

## Abstract

(1) Background: Termites are important ecosystem engineers, crucial for the maintenance of tropical biodiversity and ecosystem functioning. But they are also pests which cause billions of dollars in damage annually to humans. Currently, our understanding of the mechanisms influencing species occurrences is limited and we do not know what distinguishes pest from non-pest species. (2) Method: We analyzed how anthropogenic disturbance (agriculture) affects species occurrences. We tested the hypothesis that strong disturbance functions as a habitat filter and selects for a subset of species which are major pests of crop. Using a cross-sectional approach, we studied termite assemblage composition along a disturbance gradient from fields to 12-year-old fallows in a West African savanna. (3) Results: We reliably identified 19 species using genetic markers with a mean of about 10 species—many of them from the same feeding type—co-occurring locally. Supporting our hypothesis, disturbance was associated with environmental filtering of termites from the regional species pool, maybe via its effect on vegetation type. The most heavily disturbed sites were characterized by a subset of termite species which are well-known pests of crop. (4) Conclusion: These results are in line with the idea that strong anthropogenic disturbance selects for termite pest species.

## 1. Introduction

Termites are major ecosystem engineers with crucial roles in decomposition, soil fertility, hydrology, and species diversity [1,2]. Concomitantly, a few species are also major pests [3]. Despite their importance, we hardly understand what determines the occurrence of different termite species and what distinguishes pest from non-pest species. Niche overlap between different species seems to be substantial as termites are detritivores and only four major feeding types are distinguished [4]: Dead wood feeders (group I); dead wood, micro-epiphytes, leaf litter and grass feeders (group II); and humus feeders (group III) and true soil feeders (group IV) (reviewed in [5,6]). In African savannas up to 20 higher termite species (Termitidae) of feeding group II co-exist [7,8,9,10,11]. These group II species can be sub-divided into two feeding type specialists, grass-feeding *Trinervitermes* (group II_g_) and fungus-growing Macrotermitinae (group II_f_). The latter cultivate an obligate symbiotic fungus within their colonies, which they provision with a broad range of dead plant material [12]. The similarity of termites’ food niches implies that competitive interactions are important in shaping local savanna assemblages [13,14]. However, recent analyses suggest that random processes play an important role in species assembly in an undisturbed West African savanna, with a structuring effect by one large mound building species, *Macrotermes bellicosus* [10]. Additionally, first evidence implies that assembly processes change to more environmental filtering with disturbance [11]. This suggests that disturbance can, not only lead to a decline in species richness, but also to a change of the processes that structure species assemblages.

In the current study, we investigated termite assemblage composition of different-aged fallows (measured as the time since they were last cultivated) in a West African savanna region in Togo. By doing this, we aimed at analyzing how species assemble over time from a strong anthropogenically disturbed habitat to less degraded settings. We expect that termite assemblages of younger-aged fallows are more strongly structured by environmental filters. To test this, we first identified all species occurring in the different assemblages using morphological and genetic markers. A genetic approach is necessary to unambiguously identify all termites. To reveal assembly processes, we then applied phylogenetic community analyses that explicitly test real, studied communities against communities that are drawn at random from the regional species pool. Finally, we investigated whether species from young-aged fallows correspond to pest species.

## 2. Materials and Methods

### 2.1. Termite Sampling

**Figure 1:**
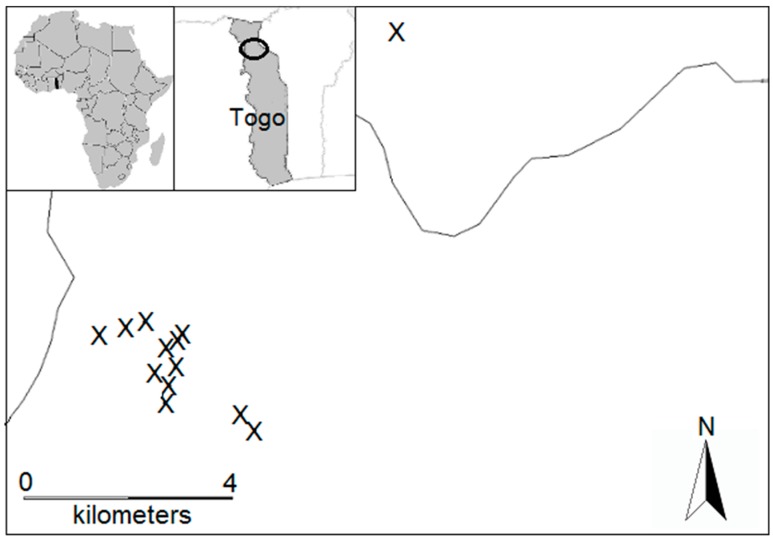
Location of the the Oti-Keran National Park in northern Togo and distribution of the 13 sampled fallows in the study area. Single lines are small roads and the double line in grey is a river.

Termites were systematically collected when they were most active, that is, during the beginning of the rainy season, near the Oti-Keran National Park in northern Togo (West Africa; 10°17′ to 10°08′ N; 0°28′ to 0°51′ E, Figure 1). This region is a typical West African savanna lying in the center of the West Sudanian biome (mean annual precipitation: 1100 mm; Worldclim database). Termites were collected in 2012 from seven fallows of age 0, 2, 4, 6, 8, 10 and 12 years. In 2014 we added six new fallows of age 0, 0, 1, 2, 10 and 10 years. Our sampling regime was constrained by the availability of fallows with a known age.

Sampling was done using a standardized belt transect protocol first developed for sampling termites in forests [15] and then adapted to savannas [9]. In short, the protocol consists of a thorough search of dead plant material on the ground, on and in trees and mounds, as well as soil sampling to assess termite diversity [15]. Plot size was one hectare with three transects each measuring 2 m × 50 m, divided into ten 2 m × 5 m sections, arbitrarily located within one plot. The three transects characterize one study plot (i.e., the number of species occurring within one hectare). Hence, our sample size is the number of plots, not the number of replicates. Each transect section was searched systematically for termites for 15 min by a trained person. Additionally, we sampled eight soil scrapes per transect section measuring 15 cm × 15 cm × 10 cm. All encountered termites were stored in 99% pure ethanol for subsequent molecular analyses.

As in the former studies [9,15,16], we chose a plot size of one hectare because the foraging ranges of termite colonies is within 100 m [16]. Hence one hectare represents the local scale where interactions between colonies occur, i.e., it reflects the Darwin-Hutchinson-Zone, which is most relevant to the study assembly of local communities [17]. We specifically selected plots with and without active *M. bellicosus* mounds as it is the main mound builder and an important ecosystem engineer, which may influence termite communities.

All samples were identified to the species level: Samples containing soldiers were first identified using the keys by Webb [18] and Sands [19], and then sequenced to obtain an unambiguous species identity (see below). Samples with workers only were genetically analyzed, as morphological identification was impossible (see below). To assign the feeding group to each sample, we followed the anatomical criteria outlined by [20]. Whenever we found/encountered termites during the search within a transect section, we collected a few specimens in a vial (5–10 individuals). Then we continued searching within the section, and when we encountered termites again, they were placed in a separate vial. The number of all resulting vials for a study plot, i.e., the sum over all transect sections for all three replicate transects within a plot, was used as the encounter rate. This is used as a surrogate of species abundance [21] (Davies 2002). The presence/absence of each species is also compiled from the same data.

During sampling, we recorded data for the environmental variable ‘vegetation type’ by classifying plots according to their vegetation type and density: Field (recognizable cultivation and crop plants), open savanna (mainly grass land, few bushes and trees), and medium-dense savanna (many bushes and trees). The savanna was a typical West African Sudanian savanna. The main shrub and tree species were *Afzelia africana*, *Crossopteryx febrifuga*, *Detarium microcarpum*, *Piliostigma thonningii*, *Vitellaria paradoxa*, *Combretum* spp., *Terminalia* spp. and *Gardenia* spp. All study plots were located away (at least 1 km) from rivers or lakes. The topography of the studied plots was flat.

### 2.2. Genetic Identification and Phylogenetic Analyses

To allow unambiguous species identification, we isolated DNA and sequenced fragments of three genes as described elsewhere [9] (additional data are given in the Supplementary Material: Table S1): *Cytochrome oxidase sub-unit I* (*COI*; total length 680 bp), *cytochrome oxidase sub-unit II* (*COII*; total length 740 bp), and *12S* (total length 350 bp). These sequences were used to re-construct phylogenetic trees using three approaches (Bayesian method, maximum-parsimony analysis, and maximum-likelihood analysis) to deliminate and identify species (for more details see Supplementary Material). As in former termite studies [9,22], *COII* was most useful for ‘barcoding’ (i.e., assigning species to samples) because it amplified well and gave appropriate resolution for species identification. All samples were identified. Species names correspond to those given in [9,10,11] for Benin. *Amitermes* sp. 1 from the Benin studies is actually *Amitermes evuncifer*. Hence, we used the proper species name in our study. To obtain corresponding species identities, we constructed a phylogeny comprising all species occurring in Togo and Benin. Samples forming a species cluster were named identically. In total we sequenced 899 samples in the current study.

### 2.3. Phylogenetic Community Structure Analyses

We analyzed the local community structure with PHYLOCOM 4.2 [23]. As the input tree for the phylogenetic community structure analyses, we used the *COII* gene tree, which was pruned prior to analysis so as to have only the species of the regional species pool included and only one representative per species in the tree. This representative was the sequence with the highest quality values for each base (maximum value of 61, multiplied by ten) as defined in Chromas 2.4.4 [24].

We calculated the net relatedness index (NRI) that measures whether locally co-occurring species are phylogenetically more/less closely related than expected by chance. It uses phylogenetic branch length to measure the distance between each sample to every other terminal sample in the phylogenetic tree, and hence the degree of overall clustering [23]. The NRI is the difference between the mean phylogenetic distance (MPD) of the tested local community and the MPD of the total community (regional) divided by the standard deviation of the latter. High positive values indicate clustering; low negative values indicate over-dispersion [25]. We tested whether our data significantly deviated from 999 random communities derived from null models using the independent swap algorithm on presence/absence data [26,27]. The swap algorithm creates swapped versions of the sample/species matrix and constrains row (species) and column (species’ presence or absence) totals to match the original matrix. The regional species pool consisted of all species from all studied localities. As suggested by [23], we used two-tailed significance tests based on the ranks that describe how often the values for the observed community were lower or higher than the random communities. With 999 randomizations, ranks equal or higher than 975 or equal and lower than 25 are significant at *p* ≤ 0.05 [28].

### 2.4. Similarity between Fallows

We quantified the compositional similarity (ß-diversity) between all localities using the Bray-Curtis sample similarity index [29], which was calculated in EstimateS version 8.2.0 [30]. It ranges from 0 to 1, with low values indicating low similarity, and high values, the reverse. The Bray-Curtis index is quantitative; the abundance of species is taken into account when calculating the shared species statistics.

### 2.5. Other Statistical Analyses

All inferential statistics were done with the statistical package SPSS 16 (IBM, Armonk, NY, USA). All tests were two-tailed. Data were tested for assumptions of parametric testing and analyses were done accordingly. For all data, qualitatively the same results (i.e., effects were significant or non-significant) were obtained when testing parametrically or non-parametrically.

## 3. Results

### 3.1. Diversity

**Figure 2:**
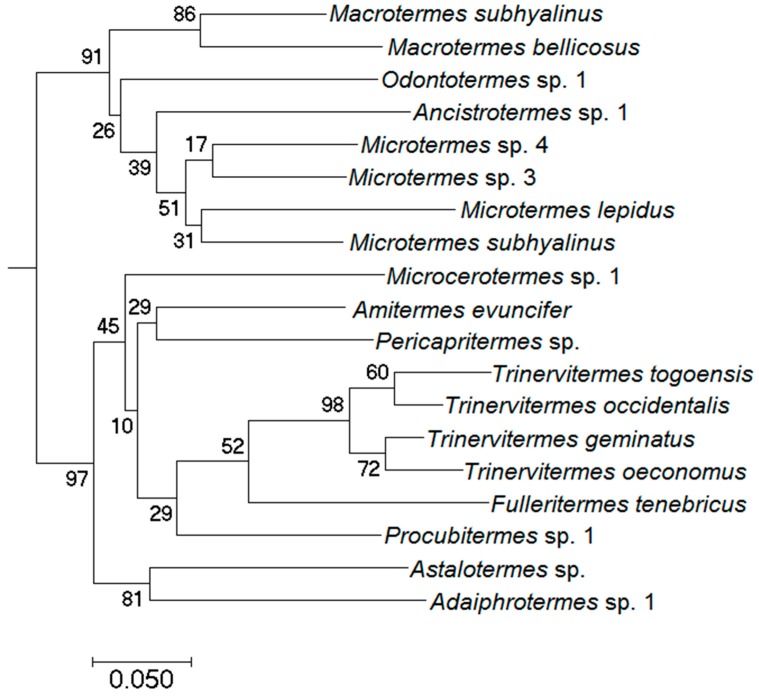
Input Bayesian phylogeny for Phylocom, based on the gene cytochrome oxidase II using MrBayes v3.1.2. Analysis was done with 10^7^ generations, number of chains = 4, sample frequency = 1000 and a finalizing burn-in of 2500. Node numbers are the posterior probabilities calculated to assess branch support.

We identified a total of 19 termite species (regional species pool), all Termitidae (Table 1). All conducted phylogenetic analyses yielded similar topologies (Figure 2; Supplementary Material: Figures S1 and S2). As is typical for African savannas, fungus-growing Macrotermitinae dominated with eight species (*Microtermes subhyalinus*, *Microtermes lepidus*, *Microtermes* sp.3, *Microtermes* sp.4, *M. bellicosus*, *M. subhyalinus*, *Ancistrotermes* sp.1, *Odontotermes* sp.1), of which all co-existed locally. The next most-species rich group were Nasutitermitinae with the grass-feeders *Trinervitermes occidentalis*, *Trinervitermes geminatus*, *Trinervitermes oeconomus*, *Trinervitermes togoensis* and *Fulleritermes tenebricus*. Despite occupying the same feeding niche, all four *Trinervitermes* species co-occurred locally. Further, we sampled two representatives of the soil-feeders Apicotermitinae (*Astalotermes* sp., *Adaiphrotermes* sp.1) and four species of the Termitinae (*Microcerotermes* sp.1, *A. evuncifer* (both wood-litter feeders)*, Procubitermes* sp.1 and *Pericapritermes* sp.1 (soil and soil-wood feeders)).

Surprisingly, species richness did not increase with fallow age (Spearman-rank correlation: *N* = 13, *p* = 0.324). Out of a total of 19 species that we found in the fallows, from seven to 13 species co-occurred locally. Species names are in accordance with the identified species from the studies in Benin [9,10,11].

### 3.2. Phylogenetic Community Structure

**Figure 3:**
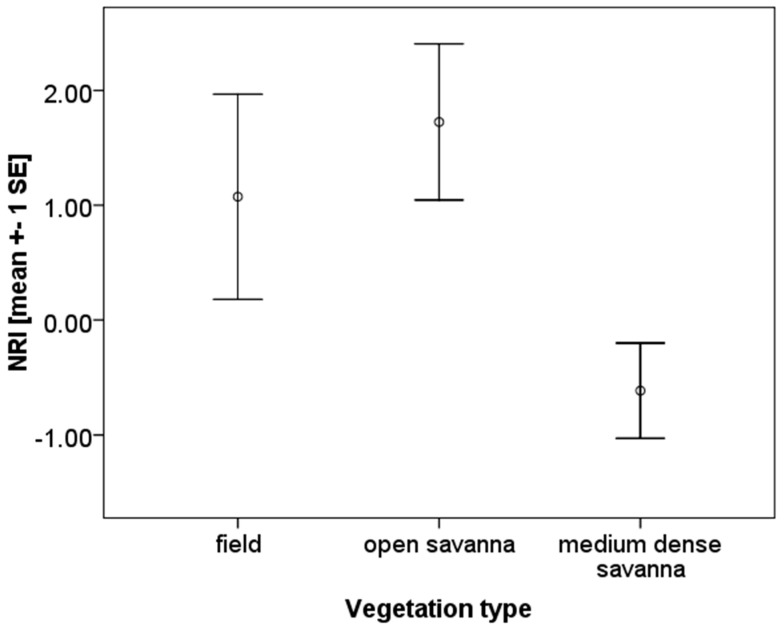
NRI (Net Relatedness Index) and vegetation type. There was an indication that vegetation patterns affect termite community composition, as communities were more clustered (more closely related) in open savannas.

The NRI values, measuring the phylogenetic community composition, ranged from −0.72 to 4.21. Three plots showed significant signals of environmental clustering (Plot S: NRI: 2.83; Plot W: NRI: 2.80; Plot 5: NRI: 4.41; all *p* < 0.05). NRI values did not correlate with fallow age (Spearman-rank correlation: *N* = 13, *p* = 0.131) nor with species richness (Spearman-rank correlation: *N* = 13, *p* = 0.890). However, there was an indication that vegetation type affects phylogenetic community structure (ANOVA: F = 3.21, *p* = 0.084, Figure 3). Communities tended to be more phylogenetically clustered in fields and especially open savannas (Turkey HSD *post-hoc* test: *Field/open*: *p* = 0.802; *field/medium dense*: *p* = 0.316; *open/medium dense*: *p* = 0.072).

**Figure 4:**
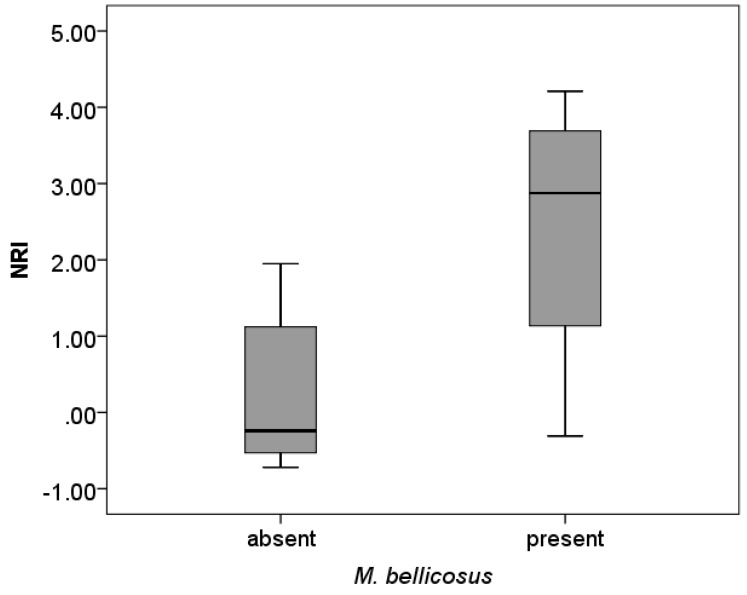
NRI and presence of *M. bellicosus*. There may be an effect of *M. bellicosus* on phylogenetic community structure. NRIs were higher (i.e., more phylogenetically clustered communities) when *M. bellicosus* was present than when it was absent (*p* = 0.079).

Similarly, *M. bellicosus* may have an effect on phylogenetic structuring: As a tendency, when *M. bellicosus* was present, NRIs were higher (i.e., more phylogenetically clustered communities) than when it was absent (Mann-Whitney-U test, Z = −1.76, *N* = 13, *p* = 0.079; Figure 4).

### 3.3. Similarity between Fallows

**Figure 5:**
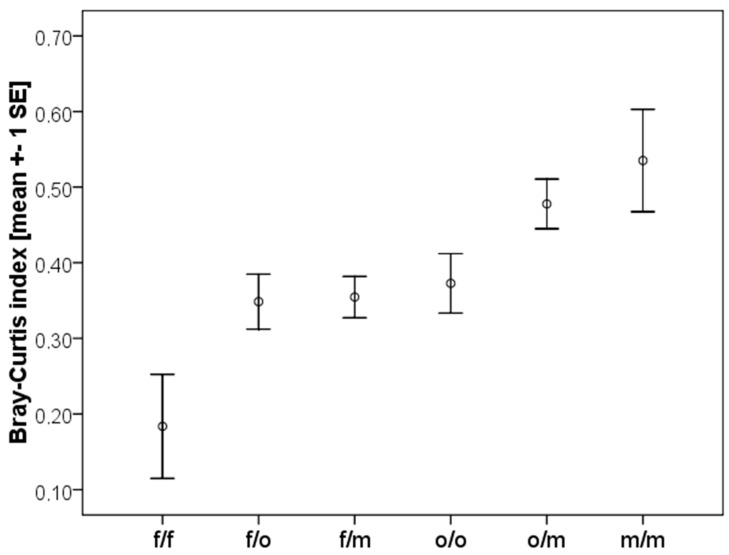
Compositional species similarity between plots of different vegetation type measured with the Bray-Curtis sample similarity index. It revealed a significant difference in species similarity between the sites of different vegetation types; f = fallow; o = open savanna; m = medium-dense savanna.

The compositional similarity between sites varied, with the Bray-Curtis index ranging from 0.075 to 0.785. Mean species richness per site was 10.1 (±SD 1.75) species and mean number of shared species between sites was 6.1 (±SD 1.64) species. When comparing sites of different vegetation types to each other, the Bray-Curtis index revealed that there is a significant difference in species composition between vegetation types (ANOVA: F = 4.329, *p* = 0.002, Figure 5). Fields (f/f) were significantly less similar among each other in species composition than open compared to medium dense savanna sites (o/m) are, or medium dense savanna sites (m/m) are among each other (Tukey-HSD *post-hoc* test: f/f vs. o/m: *p* = 0.022; f/f vs. m/m: *p* = 0.016). Open savanna sites and medium dense savanna sites had a higher species similarity among and between each other. The other compared vegetation types (f/o, f/m, o/o) lay between these extremes. Overall, there was a pattern that compositional species similarity rises, the less disturbed the sites are.

### 3.4. Pest Species

We could identify several termite species in our study sites that are known pest species [3,31,32,33]: *M. subhyalinus*, *Odontotermes* sp., *Microtermes* spp., *Pericapritermes* sp., *A. evuncifer* and *Ancistrotermes* sp. We found all these species throughout all fallows. Yet, especially *Microtermes* species, *A. evuncifer* and *Ancistrotermes* sp. were quite common in young fallows and *Pericapritermes* sp. was only sampled in young fallows (0–2 years; vegetation type ‘field’, Table 1). For *Odontotermes* sp. and *M. subhyalinus,* we did not find a specific pattern. These species occurred in young and older fallows alike, but these species were rare in general.

## 4. Discussion

Our results support the hypothesis that strong anthropogenic disturbance is associated with environmental filtering of termite species in savannas. We found no evidence that interspecific competition plays a major role in structuring these termite assemblages.

Similar to the study on termite communities of the West African savanna in Benin [11], we found a total of 19 termite species in this study in Togo, with 17 (89.47%) species shared by both areas. Despite their similar food requirements, on average 10 species co-occurred locally, all belonging to the higher termites and often sharing the same feeding and sub-feeding type. Based on a phylogenetic community analysis, we found no evidence that interspecific competition plays a major role. On the contrary, especially in strongly disturbed sites, environmental filtering seems to be important (Figure 3 and Figure 5). This confirms what was found for Benin and implies that disturbance is associated with environmental filtering in West African savanna regions, meaning that disturbance-induced habitat changes (i.e., changes in plant species composition and physical structure of the habitat) filter out certain termite species from these assemblages.

Generally, increasing disturbance is associated with a decline in species richness. This study looks at the reverse process: Increasing habitat recovery or “re-wilding” of habitats. As our results showed that species richness did not increase with fallow age (“re-wilding”), our study suggests that reducing disturbance and allowing the habitat to “re-wild” will not, by default, lead to an increase in termite species richness and re-establishment of the original termite assemblage. This result is also supported by our results of a parallel study, looking at termite assemblages in undisturbed sites in the same savanna and comparing them to the fallows discussed here. We could show that the assemblages of the two studied savanna regimes differed significantly in their compositional and phylogenetic similarity. Assemblages in the undisturbed sites are more similar to each other compositionally and phylogenetically than the ones in the fallows, and older fallows still have a different species composition than undisturbed savannah sites (unpublished data). This process can also be seen in other arthropods as well, for example in ants [34]. Also, in contrast to Benin, we did not detect a decline of species richness with disturbance [11]. Rather a higher degree of disturbance in younger fallows seems to favor a certain set of species. This difference between both studies areas may be due to the drier climate in Benin and/or because, in Benin, samples were taken next to villages with on-going land-use.

Many of the species from fields are known as crop pests in West Africa, especially *A. evuncifer*, *Ancistrotermes* sp., *Microtermes* spp. (Table 1) [3,31,32,33]. Whether the occurring termite species are pests because they are more resilient against disturbance, or whether selection as pests have made them more resilient, is difficult to test. Some of the sampled pest species also occurred in some older fallows (in particular *Odontotermes* sp. and *M. subhyalinus*) suggesting that they are generalists that can cope better with human disturbance. By contrast, grass-feeding *Trinervitermes* species (*T. togoensis*, *T. geminatus*, *T. oeconomus,* and *T. occidentalis*) were mainly found in older fallows (Table 1), implying that they are less resilient against disturbance. As grass is also commonly available in young fallows, it is unlikely that limited food availability can account for their absence. Other studies have also shown that *Trinervitermes* species are specialists concerning their feeding and nesting habits, which possibly makes them more susceptible to habitat disturbances [35,36]. Nevertheless, fields seem to be very heterogeneous among each other concerning species composition as indicated by the low Bray Curtis similarity (Figure 5). This could be due to the kind of crop being cultivated, or other biotic and abiotic factors.

As in Benin [10], several closely related fungus growers were associated with the occurrence of *M. bellicosus* (including *Microtermes*, *Ancistrotermes* sp.1 and *M. subhyalinus*), reflected in phylogenetic clustering (Figure 4). *Macrotermes* mounds can provide micro-habitats for other fungus growers, as well as facilitating their occurrence by concentrating nutrients and clay through their nest building and foraging activities [37], thereby explaining the increased phylogenetic clustering in sites with *M. bellicosus* mounds. Nevertheless, non-fungus growing termite species can also benefit from *M. bellicosus* mounds. But this does not so much seem to be the case in this savanna area.

### Comparison with Other Community Studies in West Africa

There are few studies on West African termite assemblages, and besides the above mentioned recent studies in Benin, none used a molecular approach that is necessary for unambiguous species identification of West African termites, which makes direct comparisons difficult.

Dosso et al. [8] studied termite assemblages in a more wooded region near Lamto in the Ivory Coast in land-use systems, ranging from a semi-deciduous forest, over plantations, to a crop field and a four-year old fallow. As is typical for forests [38], species richness declined—and especially soil feeders disappeared—with disturbance and a transition from forest to a more open habitat. The crop field and the four-year old fallow, which are the most comparable to our study sites, harbored 11 and 7 morpho-species, respectively. Several of these species are typical forest species and hence absent in our study (e.g., *Nasutitermes, Basidentitermes*). Only a single *Microtermes* species was found in Lamto, compared to four in this study and in Benin [9]. One species might be an under-estimation as *Microtermes* species are difficult to identify without genetic means. Strikingly, only one *Trinervitermes* species was found in Lamto, and this was in the four-year old fallow. This supports our conclusion that *Trinervitermes* spp. are more susceptible to disturbance. Another study near Lamto tested the influence of annual fires on termite diversity [7]. Here, the occurrence of *Trinervitermes* spp. in burnt areas decreased, further supporting the hypothesis that they are less resilient species.

In contrast to our study, some studies implicated evidence for inter-specific competition in structuring termite assemblages [13,14,39,40,41,42,43]. The reasons for these diverse conclusions include, differences between study sites, disturbance regimes, and lack of testing against the null hypothesis of random community assembly. Additionally, most studies focused on a few species only, and did not study whole termite assemblages, thereby addressing a different scale. More studies, spanning more regions, are necessary to derive general conclusions. Such studies should cover complete assemblages of genetically identified species where species co-/occurrences are tested against random assemblages. Genetic identification is helpful, as otherwise, especially the most closely related species may be mis-identified, which can lead to blurring signals of environmental filtering or interspecific competition.

## Figures and Tables

**Table 1:** Abundances and encounters of the 19 species of the regional species pool in all study plots including data on fallow age and vegetation type.

	F	sf	fg	L	M	N	O	P	R	S	T	U	W	2	3	5
*Trinervitermes occidentalis*	Te	N	II_g_	15	2	2	1	5	5	3	8	9	0	1	0	0
*Trinervitermes geminatus*	Te	N	II_g_	3	2	4	0	7	6	6	2	13	0	1	0	0
*Trinervitermes oeconomus*	Te	N	II_g_	5	6	2	0	2	0	3	1	3	0	4	1	0
*Trinervitermes togoensis*	Te	N	II_g_	8	2	6	0	6	12	13	0	2	0	1	2	0
*Fulleritermes tenebricus*	Te	N	II	0	0	0	0	0	0	0	0	1	0	1	0	0
*Microtermes subhyalinus*	Te	M	II_f_	0	2	1	4	2	3	0	0	0	3	5	0	2
*Microtermes lepidus*	Te	M	II_f_	1	0	4	5	1	0	0	6	6	13	2	1	6
*Microtermes* sp.3	Te	M	II_f_	4	10	3	3	4	0	2	1	0	2	5	5	2
*Microtermes* sp.4	Te	M	II_f_	1	0	3	8	0	1	0	1	1	4	2	0	2
*Macrotermes bellicosus*	Te	M	II_f_	0	2	0	2	0	0	0	0	2	0	5	0	18
*Macrotermes subhyalinus*	Te	M	II_f_	0	1	0	0	0	2	0	0	0	0	0	0	3
*Ancistrotermes* sp.1	Te	M	II_f_	0	0	0	25	0	0	0	0	7	19	28	10	16
*Odontotermes* sp.1	Te	M	II_f_	0	0	1	0	0	0	0	9	9	3	0	0	1
*Astalotermes* sp.	Te	Ap	III	4	2	1	0	0	3	3	0	1	1	0	0	0
*Adaiphrotermes* sp.1	Te	Ap	III	0	0	2	1	1	0	0	1	4	0	0	1	2
*Microcerotermes* sp.1	Te	T	II	6	0	11	13	11	17	23	33	15	5	14	15	2
*Amitermes evuncifer*	Te	T	II	0	0	0	1	0	3	0	5	0	13	0	5	0
*Procubitermes* sp.1	Te	T	IV	0	0	0	0	0	0	0	1	0	0	1	0	0
*Pericapritermes* sp.	Te	T	III	0	1	0	0	0	0	0	0	0	0	0	1	0
Number of species				9	10	12	10	9	9	7	11	13	9	13	9	10
Number of encounters				47	30	40	63	39	52	53	68	73	63	70	41	54
Fallow age (years)				8	0	6	2	4	10	12	0	10	2	1	1	10
Vegetation type				1	0	2	1	2	2	1	0	1	0	2	1	1

Shown are encounters and number of species per study plot together with feeding groups (fg). Plots L, M, N, O, P, R, S were sampled in 2012; T, U, W, 2, 3 and 5 were sampled in 2014. f: family; Termitidae (Te); sf: subfamily: Macrotermitinae (M), Nasutitermitinae (N), Termitinae (T), Apicotermitinae (Ap). The classification of feeding groups follows Donovan et al. (2001): I: Dead wood-feeders; II: Wood-litter feeders (II_g_: Grass feeders; II_f_: Fungus growers); III: Humus feeders; IV: *True* soil feeders. The classification of vegetation types are: 0 = field, 1 = open, 2 = medium dense.

## Supplementary Materials

The following are available online at http://www.mdpi.com/2075-4450/10/1/17/s1, Figure S1: Bayesian phylogeny based on the gene cytochrome oxidase I using MrBayes v3.1.2; Figure S2: Bayesian phylogeny based on the ribosomal gene 12S using MrBayes v3.1.2; Table S1: Primers with sequences and annealing temperatures for the genes *COI*, *COII*, *12S*.
