# Supplementary material for: Termite Communities along A Disturbance Gradient in a West African Savanna
Source: Insects. 2019 Jan 8;10(1):17. doi: 10.3390/insects10010017 (PMC6358944; doi:10.3390/insects10010017)
Supplement: Supplementary file 1 [file insects-10-00017-s001.pdf]

Termite Communities along A Disturbance Gradient in a West African Savanna

Janine Schyra [1,*] and Judith Korb [1,2]

[1] Behavioral Biology, University of Osnabrueck, Barbarastr. 11, D-49076 Osnabrueck, Germany; judith.korb@biologie.uni-freiburg.de
[2] Evolution and Ecology, Albert-Ludwigs-University Freiburg, Hauptstr. 1, D-79104 Freiburg im Breisgau, Germany
* Correspondence: Janine.Schyra@Biologie.Uni-Osnabrueck.de;
Tel.: +49 (0)351-795841-4359; Fax: +49 (0)351 795841-4444

Genetic and phylogenetic analyses

DNA was isolated from the head of single individuals using a modified cetyltrimethyl ammonium bromide (CTAB)-protocol as described in Fuchs et al. (2003) [1].

The gene *COII* was amplified using the primer pair Modified A-tLeu and B-tLys, *COI* was amplified using the primers HCO and LCO and the ribosomal gene *12S* was amplified using 12Sai_for/12Sbi_rev (Table S1). PCR were performed with the following cycle conditions for *COI* and *COII*: 94 °C for 2 min; and then 35 cycles of 94 °C for 1 min, 50 °C for 1 min, 72 °C for 1 min 15 s and a final elongation step of 72 °C for 7 min. For *12S* the cycle conditions were the same except for the annealing temperature, which was 55 °C. PCR amplifications were purified using poly ethylene glycol (PEG) mix and sequencing was performed using BigDye Terminator v3.1 (concentration of 2:1, Applied Biosystems, Foster City, CA, USA) with cycle sequencing conditions of 96 °C for 1 min, then 30 cycles of 96 °C for 30 s, 50 °C for 15 s, and 60 °C for 4 min on an ABI 3500 Genetic Analyser (Applied Biosystems).

To identify species, sequences for each gene were aligned separately using BioEdit [2] and checked visually for missing or false bases at the nucleotide- as well as the amino acid level. Analyses were performed for each gene as in Hausberger et al. [3]. In short, we inferred phylogenies using (i) a Bayesian method with MrBayes (Huelsenbeck and Ronquist [4]) (10^7 generations, 25% discarded as burn-in), (ii) a maximum parsimony analysis (MP) with PAUP 4.0 [5] (heuristic search with 100 random addition replicates from random starting trees with TBR (tree bisection reconnection)), and (iii) a maximum-likelihood (ML) analysis using RaxML [6]. Nucleotide substitution models were selected with MrModeltest 2.3 [7]. Posterior probabilities (Bayesian inference), decay values (MP) and bootstrap values (ML) were calculated to assess branch support.

Table S1. Primers with sequences and annealing temperatures for the genes *COI, COII, 12S*.

Gene	Primer	Sequence 5′-3′	Annealing Temperature	Reference
COI	HCO	TAA ACT TCA GGG TGA CCA AAA AAT CA	50 °C	[8]
	LCO	GGT CAA CAA ATC ATA AAG ATA TTG G	50 °C	[8]
COII	Modified A-tLeu	CAG ATA AGT GCA TTG GAT TT	50 °C	[9]
	B-tLys	GTT TAA GAG ACC AGT ACT TG	50 °C	[9]
12S	12Sai_for	AAA CTA GGA TTA GAT ACC CTA TTA T	55 °C	[10]
	12Sbi_rev	AAG AGC GAC GGG CGA TGT GT	55 °C	[10]

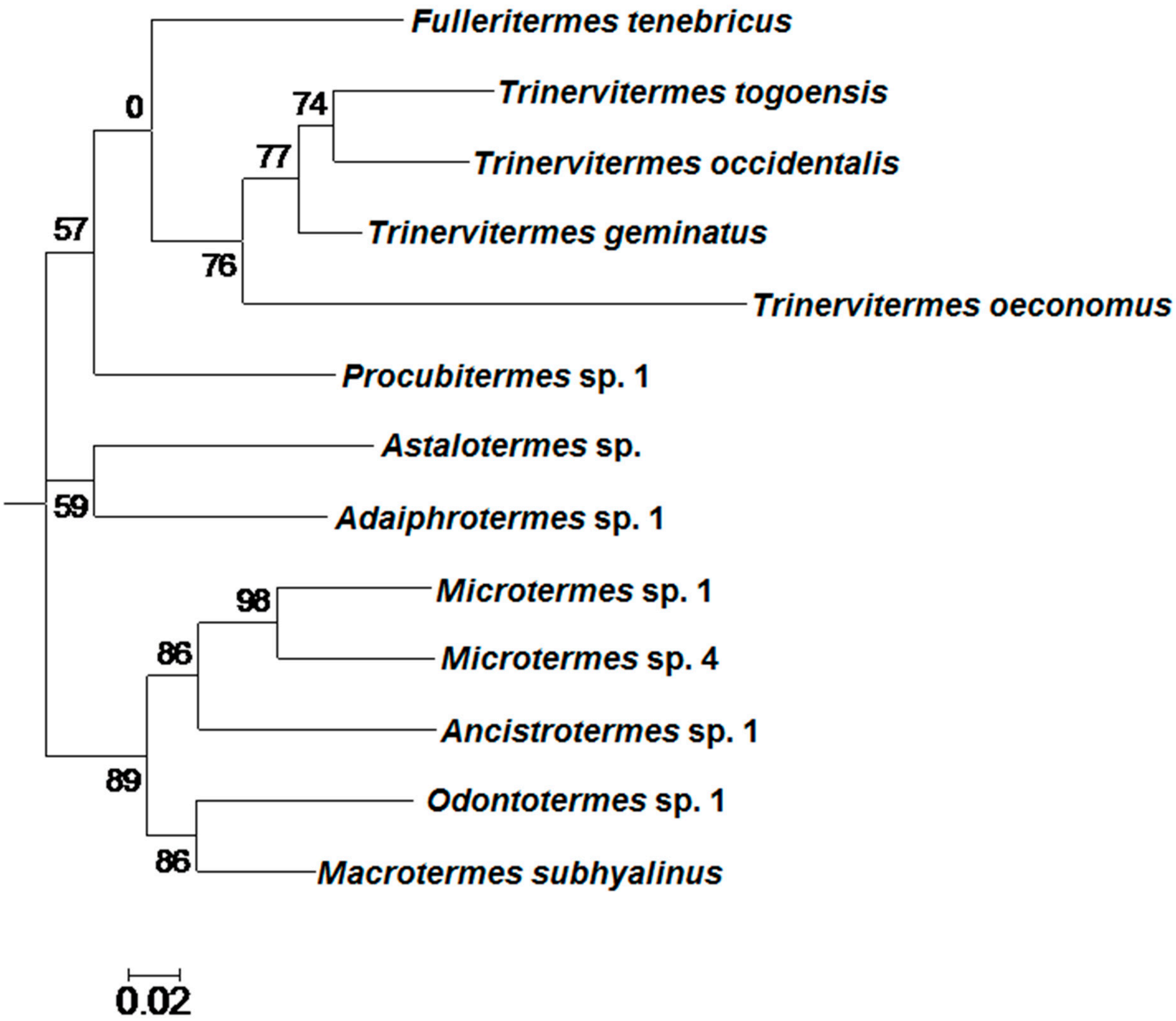

Figure S1. Bayesian phylogeny based on the gene cytochrome oxidase I using MrBayes v3.1.2. Analysis was done with 10^7 generations, number of chains = 4, sample frequency = 1000 and a finalizing burn-in of 2500. Due to primer binding problems during amplification, not all species are included. Numbers on nodes are the posterior probabilities calculated to assess branch support.

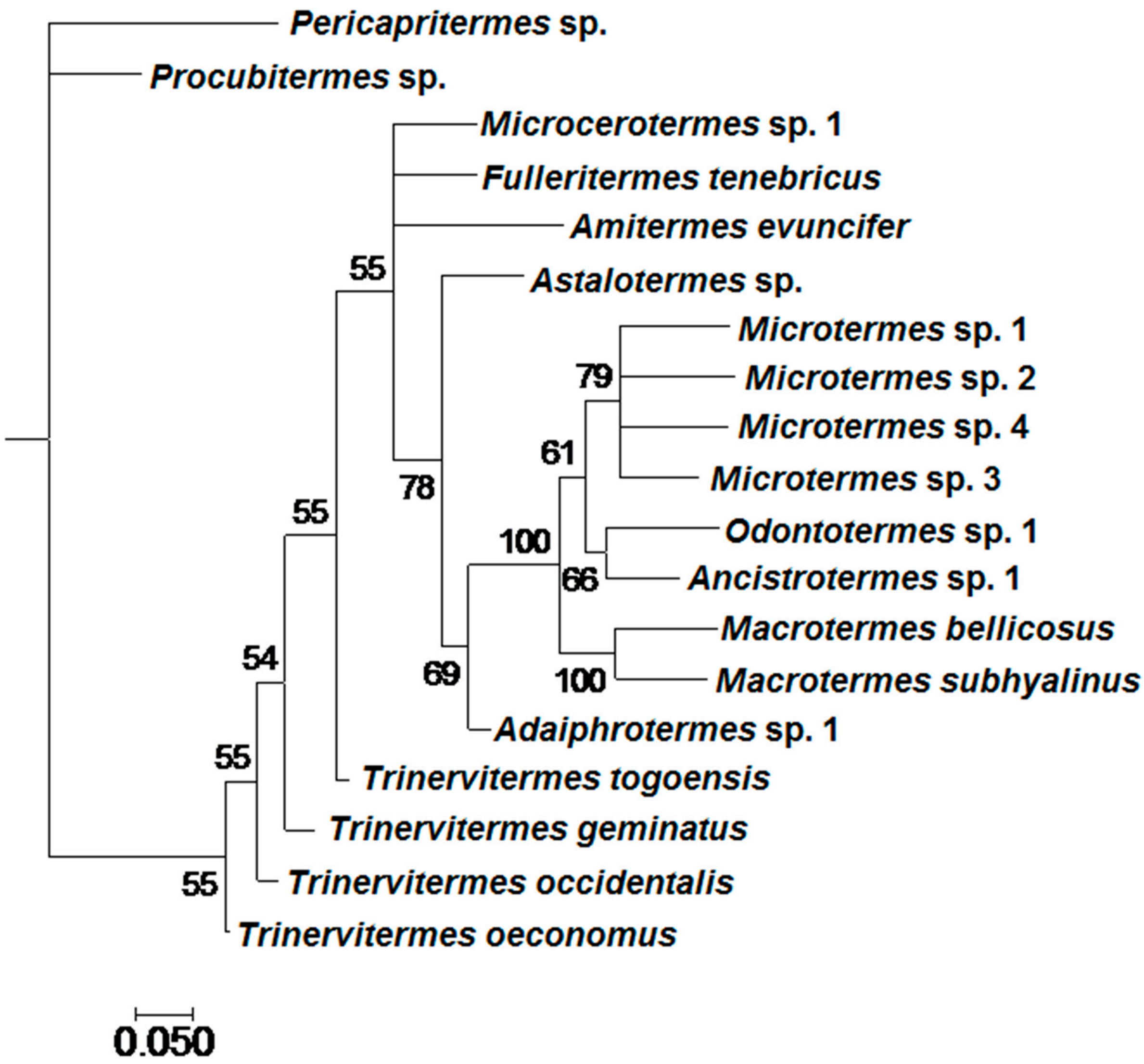

Figure S2. Bayesian phylogeny based on the ribosomal gene *12S* using MrBayes v3.1.2. Analysis was done with 10^7 generations, number of chains = 4, sample frequency = 1000 and a finalizing burn-in of 2500. Due to primer binding problems during amplification, not all species are included. Numbers on nodes are the posterior probabilities calculated to assess branch support.

Reference

1. Fuchs, A.; Heinze, J.; Reber-Funk, C.; Korb, J. Isolation and characterization of six microsatellite loci in the drywood termite Cryptotermes secundus (Kalotermitidae). *Mol. Ecol. Notes* **2003**, *3*, 355–357.
2. Hall, T.A. BioEdit: A user-friendly biological sequence alignment editor and analysis program for Windows 95/98/NT. *Nucleic Acids Symp. Ser.* **1999**, *41*, 95–98.
3. Hausberger, B., Kimpel, D.; van Neer, A.; Korb, J. Uncovering cryptic species diversity of a community in a West African savanna. *Mol. Phylogenet. Evol.* **2011**, *61*, 964–969.
4. Huelsenbeck, J.P.; Ronquist, F. MrBayes3: Bayesian phylogenetic inference under mixed models. *Bioinformatics* **2003**, *19*, 1572–1574.
5. Swofford, D.L. PAUP*: Phylogenetic analysis using parsimony (and Other Methods). Sinauer Associates: Sunderland, MA, USA, 1998.
6. Stamatakis, A. RAxML-VI-HPC: Maximum likelihood-based phylogenetic analyses with thousands of taxa and mixed models. *Bioinformatics* **2006**, *22*, 2688–2690.

7. Nylander, J.A.A. MrModeltest version 2. Program distributed by the author. Evolutionary Biology Centre, Uppsala University, 2004. Avaiable online: https://github.com/nylander/MrModeltest2 (accessed on 1 May 2012).
8. Folmer, O.; Black, M.; Hoeh, W.; Lutz, R.; Vrijhoek, R. DNA primers for amplification of mitochondrial cytochrome c oxidase subunit I from diverse metazoan invertebrates. *Mol. Mar. Biol. Biotech.* **1994**, *3*, 294–299.
9. Inward, D.J.G.; Vogle, A.P.; Eggleton, P. A comprehensive phylogenetic analysis of termites (Isoptera) illuminates key aspects of their evolutionary biology. *Mol. Phylogenet. Evol.* **2007**, *44*, 953–967.
10. Simon, C.; Frati, F.; Beckenbach, A.; Crespi, H.C.; Flook, P. Evolution, weighting, and phylogenetic utility of mitochondrial gene sequences and a compilation of conserved polymerase chain reaction primers. *Ann. Entomol. Soc. Am.* **1994**, *87*, 651–701.
